# HLA-DRB1 Alleles Are Associated with the Susceptibility to Sporadic Parkinson’s Disease in Chinese Han Population

**DOI:** 10.1371/journal.pone.0048594

**Published:** 2012-11-06

**Authors:** Congcong Sun, Lei Wei, Feifei Luo, Yi Li, Jiaobiao Li, Feiqi Zhu, Ping Kang, Rensi Xu, LuLu Xiao, Zhuolin Liu, Pingyi Xu

**Affiliations:** 1 Department of Neurology, The First Affiliated Hospital of Sun Yat-sen University, Guangzhou, People’s Republic of China; 2 Department of Neurology, The Third Affiliated Hospital of Sun Yat-sen University, Guangzhou, People’s Republic of China; 3 Department of Neurology, The Third People’s Hospital of Chengdu, Chengdu, People’s Republic of China; 4 Department of Neurology, Department of Neurology, Red-Cross Hospital in Guangzhou, Guangzhou, People’s Republic of China; 5 Department of Neurology, HeYuan City Hospital, Guangdong, People’s Republic of China; 6 Department of Neurology, Yuebei People's Hospital of Shantou University Medical College, Guangdong, People’s Republic of China; 7 Department of Neurology, The First People’s Hospital of Shaoguan, Guangdong, People’s Republic of China; 8 Department of Neurology, The First Affiliated Hospital of NanChang University, NanChang, People’s Republic of China; 9 Department of Tissue Typing Center, Nanfang Hospital of Southern Medical University, Guangzhou, People’s Republic of China; National Institutes of Health, United States of America

## Abstract

Immune disorders may play an important role in the pathogenesis of Parkinson's disease (PD). Recently, polymorphisms in the HLA-DR region have been found to be associated with sporadic PD in European ancestry populations. However, polymorphisms in the HLA complex are highly variable with ethnic and geographic origin. To explore the relationships between polymorphisms of the HLA-DR region and sporadic PD in Chinese Han population, we genotyped 567 sporadic PD patients and 746 healthy controls in two independent series for the HLA-DRB1 locus with Polymerase chain reaction-sequence based typing(PCR-SBT). The χ^2^ test was used to evaluate the distribution of allele frequencies between the patients and healthy controls. The impact of HLA-DRB1 alleles on PD risk was estimated by unconditional logistic regression. We found a significant higher frequency of HLA-DRB1*0301 in sporadic PD patients than in healthy controls and a positive association, which was independent of onset age, between HLA-DRB1*0301 and PD risk. Conversely, a lower frequency of HLA-DRB1*0406 was found in sporadic PD patients than in healthy controls, with a negative association between HLA-DRB1*0406 and PD risk. Furthermore, a meta-analysis involving 195205 individuals was conducted to summarize the frequencies of these two alleles in populations from various ethnic regions, we found a higher frequency of HLA-DRB1*0301, but a lower frequency of HLA-DRB1*0406 in European ancestry populations than that in Asians, this was consistent with the higher prevalence of sporadic PD in European ancestry populations. Based on these results, we speculate that HLA-DRB1 alleles are associated with the susceptibility to sporadic PD in Chinese Han population, among them HLA-DRB1*0301 is a risk allele while the effect of HLA-DRB1*0406 deserves debate.

## Introduction

Parkinson’s disease (PD), the second most common human neurodegenerative disease, is characterized by progressive degeneration of dopaminergic neurons in the substantia nigra (SN) and other brainstem nuclei [1]. To date, the exact etiology of sporadic PD still remains unknown. Immune dysfunctions have been confirmed to be one of the causes of this disease [2]–[4]. In the brain of PD patients, large numbers of microglia expressing human leucocyte antigen (HLA)-DR have been detected in the SN, particularly in areas of maximal neurodegeneration [5]. The HLA-DR antigen, encoded by HLA-DRA and HLA-DRB alleles, belongs to HLA class II molecules and acts as an antigen-presenting molecule or regulatory molecule involved in the specific immune response and innate immune response [6]. The highly polymorphic HLA complex plays an important role in genetic susceptibility to human diseases, and different alleles of HLA loci are responsible to variable immune responses among individuals [7].

Associations between genotype of HLA loci and degenerative diseases in the central nerve system (CNS) have been found in many populations. For example, the association between HLA-A*01, A*2402, HLA-DRB1*03 or DRB1*1501 and high risk for Alzheimer disease has been reported [8]–[11]. Possible associations of HLA-A*2 and A*28, HLA-B*17 and B*18, and HLA-DQB1*06 with PD have also been reported [12]–[15]. However, in those studies, the enrolled subjects were relatively small by low-resolution genotyping analysis, and therefore, the conclusions could be ambiguous. Recently, Saiki *et al.* found that HLA-DRB1*03 was more common among British PD patients than healthy controls with low-resolution typing [16]. In addition, large-scale genome-wide association studies indicated that genetic susceptibility loci for late-onset sporadic PD might have existed in the HLA-DR region, such as rs3129882 in HLA-DRA and chr6∶32588205 in HLA-DRB5 [17]–[19]. These studies suggest that the HLA-DR region is susceptive to the development of PD in European ancestry populations.

However, the HLA complex is highly polymorphic with different ethnic and geographic origins, PD genome-wide association studies (GWAS) study in Japanese shown no evidence of HLA association [20], while the relationship between HLA-DR polymorphism and PD in Chinese Han population has not yet been extensively investigated. Because HLA-DRB1 is the most polymorphic locus in the HLA-DR region [21], we systematically analyzed the polymorphism of HLA-DRB1 alleles among sporadic PD patients and healthy controls from a Han population living in the Guangdong province of the People’s Republic of China (PRC) through polymerase chain reaction-sequencing based typing (PCR-SBT). The potential association of HLA-DRB1 alleles with PD in this population was evaluated, and gender and the onset age were taken into account.

## Materials and Methods

### Patients

A total of 567 sporadic PD patients and 746 unrelated healthy controls of Han population root from Guangdong province of the PRC were enrolled for this study(Basic information shown in Table 1). Each subject signed an informed consent before participating. The Ethics Committee of the First Affiliated Hospital of Sun Yat-sen University approved the protocol for this study. All PD patients were diagnosed according to the modified UK Brain Bank criteria [22] at the Parkinson Clinic Center in the First Affiliated Hospital of Sun Yat-sen University from 2007 to 2012. The healthy controls were randomly selected from blood donor volunteers in the health examination center of The First Affiliated Hospital of Sun Yat-sen University. The average age of the PD patients and healthy controls was 62.20±19.10 years (range: 16–88) and 58.52±14.30 years (range: 18–85) respectively.

**Table 1:** Basic information of patients with sporadic Parkinson’s disease (PD) and healthy controls.

Sample size	Series1		Series2		Combination	
	PD	Control	PD	Control	PD	Control
**Total**	374	503	193	243	567	746
**Onset age≤50** **(Male/Female)**	74(31/43)	113(76/37)	33(11/22)	58(20/38)	107(42/65)	171(96/75)
**Onset age>50** **(Male/Female)**	300(177/123)	390(240/150)	160(66/94)	185(75/110)	460(243/217)	575(315/260)

### DNA Extraction and HLA Typing

Whole blood samples were collected from PD patients and healthy controls. Genomic DNA was extracted from 4 ml of whole blood using the QIA-amp DNA Mini Kit (QIAGEN, German) according to the manufacturer’s protocols. Extracted genomic DNA samples were stored at −80**°**C until genotyping was performed. The primer pairs recommended by the International Histocompatibilty Working Group (IHWG) were used to subtype the alleles of HLA-DRB1. HLA-DRB1 allele groups were typed by polymerase chain reaction-sequencing based typing (PCR-SBT) described previously [23]. Briefly, 22 group-specific primer pairs were used to amplify exon 2 of HLA-DRB1 using a thermal cycler (model 9700, Applied Biosystems, Foster City, CA, USA). PCR products were purified by incubating them with exonuclease at 37**°**C for 30 min and then with shrimp alkaline phosphatase (SAP) at 80**°**C for 15 min. We sequenced each purified PCR product using the ABI BigDye Terminatorv3.1 Cycle Sequencing Kit (Applied Biosystems, USA) and the ABI Prism 3100 Genetic Analyzer (Applied Biosystems). The sequence data were analyzed using the Match Tools and Navigator software (Match Tools Allele Identification package, Applied Biosystems, USA).

### Statistic Analyses

Alleles were tested for deviation from Hardy-Weinberg equilibrium (HWE) using the SHEsis software platform [24]. Phenotype Frequency was defined as the total number of carriers in the population(Carriers/n), Allele Frequency was defined as the total number of copies of the allele in the population sample (Alleles/2n). The χ^2^ test was used to evaluate the distribution of allele frequencies between the patients and healthy controls, The Fisher exact test was used when expected frequencies were less than five, In addition, Bonferroni adjusted *p* values (*p*c) were used to avoid an alpha inflation when chaining statistical tests, the number of chaining statistical tests was defined as the number of the detected alleles. The association between PD and alleles was represented by odds ratio (OR), which was estimated by unconditional logistic models adjusted for the age of onset and gender. Statistical power of every allele can be calculated by the formula “
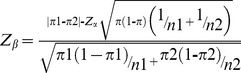
_,_ 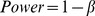 “, π1 = the phenotype frequency of the allele in group1, π2 = the phenotype frequency of the allele in group2, n1 = the sample size of group1, n2 = the sample size of group2, 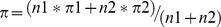, α = 0.05, Z_α/2_ = 1.960 (two sides). Calculations were carried out using SPSS-13.0 software (SPSS Inc., Chicago, IL). A *p*c<0.05, statistic Power>0.75 was regarded as statistically significant.

**Table 2:** Frequencies of HLA-DRB1 phenotypes and alleles in patients with Parkinson’s disease (PD) and healthy controls.

HLA-DRB1 Alleles	Series 1			Series 2			CombinationP/Pc
	PDN = 374	ControlN = 503	P/Pc	PDN = 193	ControlN = 243	P/Pc	
**0101**	4(0.0053)	12(0.0119)	ns	–	4(0.0082)	ns	
**0102**	–	2(0.0020)	ns	–	–	ns	
**0301**	63(0.0869)	48(0.0477)	0.001/0.041	27(0.0699)	18(0.0391)	0.025/ns	9.728E-5/4.572E-3
**0304**	–	1(0.0010)	ns	–	–	ns	
**0401**	3(0.0040)	10(0.0099)	ns	2(0.0052)	7(0.0144)	ns	
**0402**	–	1(0.0010)	ns	–	–	ns	
**0403**	23(0.0307)	16(0.0159)	0.035/ns	15(0.0389)	9(0.0185)	ns	0.005/ns
**0404**	14(0.0187)	11(0.0109)	ns	6(0.0155)	3(0.0062)	ns	
**0405**	38(0.0508)	47(0.0467)	ns	18(0.0466)	26(0.0535)	ns	
**0406**	1(0.0013)	30(0.0308)	8.036E-7/3.295E-5	3(0.0078)	12(0.0267)	0.097/ns	3.229E-6/1.518E-4
**0407**	–	1(0.0010)	ns	–	–	ns	
**0410**	–	1(0.0010)	ns	–	1(0.0021)	ns	
**0701**	36(0.0495)	53(0.0567)	ns	19(0.0570)	38(0.0823)	ns	
**0702**	–	2(0.0020)	ns	–	–	ns	
**0801**	–	2(0.002)	ns	–	–	ns	
**0802**	3(0.0040)	5(0.0050)	ns	–	1(0.0021)	ns	
**0803**	53(0.0749)	70(0.0716)	ns	31(0.0881)	36(0.0802)	ns	
**0809**	1(0.0013)	5(0.0050)	ns	–	–	ns	
**0901**	115(0.1618)	159(0.167)	ns	57(0.1580)	84(0.1831)	ns	
**1001**	7(0.0094)	16(0.0159)	ns	3(0.0078)	7(0.0144)	ns	
**1101**	34(0.0455)	50(0.0507)	ns	19(0.0492)	21(0.0432)	ns	
**1104**	–	2(0.0020)	ns	1(0.0026)	–	ns	
**1106**	2(0.0027)	–	ns	–	–	ns	
**1201**	25(0.0334)	46(0.0497)	ns	10(0.0259)	18(0.0370)	ns	
**1202**	80(0.1123)	81(0.0875)	0.045/ns	33(0.0907)	41(0.0885)	ns	ns
**1218**	3(0.0040)	–	ns	–	–	ns	
**1220**	–	–	ns	–	1(0.0021)	ns	
**1301**	2(0.0027)	8(0.0080)	ns	4(0.0104)	2(0.0041)	ns	
**1302**	17(0.0227)	34(0.0348)	ns	10(0.0259)	16(0.0329)	ns	
**1305**	–	1(0.0010)	ns	–	–	ns	
**1312**	14(0.0187)	8(0.0080)	0.044/ns	7(0.0181)	5(0.0103)	ns	0.027/ns
**1403**	3(0.0040)	4(0.0040)	ns	1(0.0026)	3(0.0062)	ns	
**1404**	4(0.0053)	8(0.0080)	ns	3(0.0078)	3(0.0062)	ns	
**1405**	21(0.0281)	19(0.0189)	ns	12(0.0311)	10(0.0206)	ns	
**1406**	–	–	ns	–	1(0.0021)	ns	
**1407**	–	2(0.0020)	ns	–	2(0.0041)	ns	
**1410**	–	–	ns	–	1(0.0021)	ns	
**1418**	–	4(0.0040)	ns	–	–	ns	
**1422**	–	1(0.0010)	ns	–	–	ns	
**1454**	28(0.0428)	21(0.0209)	0.035/ns	15(0.0389)	13(0.0288)	ns	0.021/ns
**1501**	75(0.1016)	111(0.1183)	ns	42(0.1192)	50(0.1111)	ns	
**1502**	27(0.0361)	33(0.0328)	ns	12(0.0311)	13(0.0267)	ns	
**1504**	–	1(0.0010)	ns	1(0.0026)	–	ns	
**1545**	–	–	ns	–	1(0.0021)	ns	
**1601**	–	–	ns	–	1(0.0021)	ns	
**1602**	30(0.0414)	41(0.0427)	ns	19(0.0492)	19(0.0391)	ns	

Phenotype Frequency (Allele Frequency) was presented in every cell. “−”: the allele was not been detected. Phenotype Frequency: Percentage of individuals who have the allele (Individuals/N) in percentage format. Allele Frequency: Total number of copies of the allele in the population sample (Alleles/2N) in decimal format, Pc = correction of P value (Bonferroni adjustment), Pc<0.05 is considered as significant, ns = not significant. Patients had significant higher frequencies of HLA-DRB1*0301 and lower frequency of HLA-DRB1*0406 than healthy controls did.

## Results

### The Distribution of HLA-DRB1 Alleles between PD Patients and Healthy Controls

HLA-DRB1 genotype frequencies in both PD patients and healthy controls in two series were remained constant according to HWE. A total of 46 HLA-DRB1 alleles were detected in all studied individuals, among them 41 alleles were detected in series 1, 34 alleles were detected in series 2.

We found that PD patients presented higher allele frequencies of HLA-DRB1*0301 (0.0869 vs. 0.0477, *p* = 0.001 in series 1, 0.0699 vs. 0.0391, p = 0.025 in series 2, 0.0811 vs. 0.0449, p = 9.728E-5 in combined series 1 and series 2), DRB1*0403 (0.0307 vs. 0.0159, *p* = 0.035 in series 1), DRB1*1202 (0.1123 vs. 0.0875, *p* = 0.045 in series 1), DRB1*1312 (0.0187 vs. 0.0080, *p* = 0.044 in series 1), and DRB1*1454 (0.0428 vs. 0.0209, *p* = 0.035 in series 1) than the healthy controls. Conversely, the HLA-DRB1*0406 was found with lower allele frequency in PD patients compared to healthy controls (0.0013 vs. 0.0308, *p* = 8.036E-7 in series 1, 0.0078 vs. 0.0267, p = 0.097 in series 2, 0.0035 vs. 0.0295, p = 3.229E-6 in combined series 1 and series 2). However, after Bonferroni adjustment only the differences of distribution of the HLA-DRB1*0301 (*p*c = 0.041 in series 1, pc = 4.572E-3 in combined series 1 and series 2) and HLA-DRB1*0406 (*p*c = 3.295E-5 in series 1, pc = 1.518E-4 in combined series 1 and series 2) alleles were statistically significant (statistic power >0.75) (Table 2). Using unconditional logistic regression analysis adjusted for the age of onset and gender, we found that the HLA-DRB1*0301 allele was positively associated with the risk of PD (OR = 1.963, *p* = 0.001 in series 1, OR = 2.135, p = 0.019 in series 2, OR = 2.048, p = 3.952E-5 in combined series 1 and series 2), while the HLA-DRB1*0406 allele might be negatively associated with the risk of PD (OR = 0.043, *p* = 0.002 in series 1, OR = 0.312, p = 0.077 in series 2, OR = 0.118, p = 5.002E-5 in combined series 1 and series 2).

**Figure 1:**
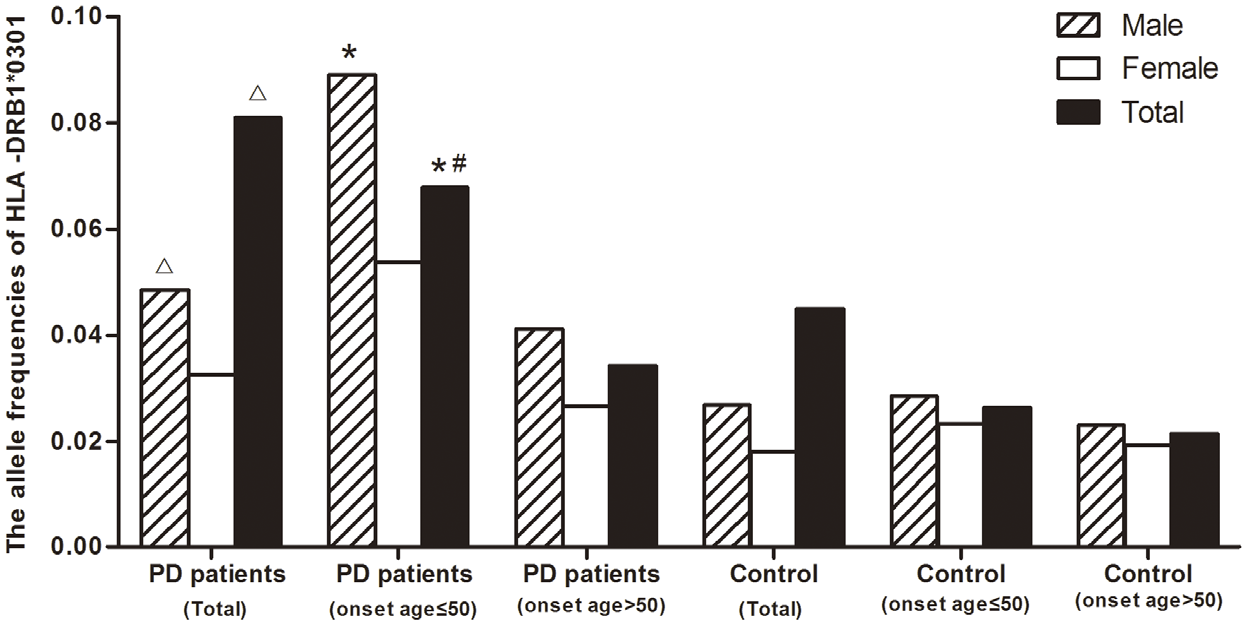
Comparison of the allele frequencies of HLA-DRB1*0301 among various groups. All subjects were classified by gender and age of onset of Parkinson’s disease (PD). △Pc<0.05 the allele frequency of HLA-DRB1*0301 in PD patients(total) vs. the one in healthy control(total), *Pc<0.05 the allele frequency of HLA-DRB1*0301 in PD patients (onset age ≤50) vs. the one in healthy control subgroup (onset age ≤50), # Pc<0.05 the allele frequency of HLA-DRB1*0301 in PD patients (onset age ≤50) vs. the one in PD patients (onset age >50).

### The Relationship between HLA-DRB1*0301 and PD Risk

To further analyze the relationship between HLA-DRB1*0301 and PD risk, we compared the allele frequencies of HLA-DRB1*0301 between subgroups classified by the gender and the onset age of all subjects through 20 independent χ2 tests and statistic power calculation described previously. We found that the allele frequencies of HLA-DRB1*0301 were higher in both PD subgroups (onset age ≤50 and onset age >50) than in their healthy control subgroups (0.0678 vs. 0.0263, *p* = 0.001, 0.0342 vs. 0.0213, p = 0.008, respectively), these differences were only occurred in male subgroups (0.0892 vs. 0.0286, *p* = 0.001, 0.0412 vs. 0.0230, p = 0.014, respectively). More interestingly, we found that the allele frequency of HLA-DRB1*0301 was higher in patients with an onset age ≤50 than in patients with an onset age >50 (0.0678 vs. 0.0342, *p = 0.001* for all patients, 0.0892 vs. 0.0412, *p = 0.003* for male patients and 0.0538 vs. 0.0265, *p = 0.046* for female patients). After Bonferroni adjustment, the differences of allele frequency between PD subgroups (onset age ≤50) and healthy control subgroups (onset age ≤50) and PD subgroups (onset age >50) was statistically significant(Pc = 0.020 and Pc = 0.020, statistic Power >0.75, respectively)(Fig.1). Using stratification analyses and unconditional logistic regression analysis adjusted for gender, we found that the positive association between HLA-DRB1*0301 and PD risk was independent of onset age(onset age ≤50:OR = 3.350, P = 4.039E-4, onset age >50: OR = 1.726, P = 0.007).

**Figure 2:**
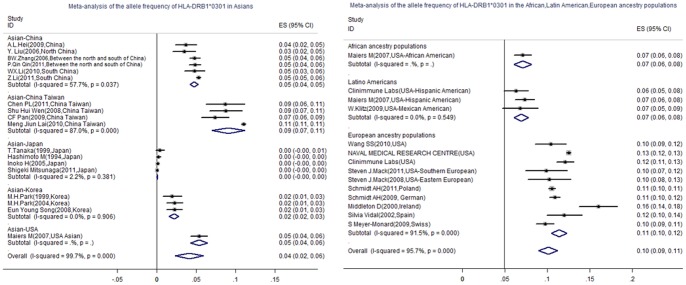
Forest plots summarizing the Allele frequency of HLA-DRB1*0301 in Asian and European ancestry populations from various regions. The allele frequency was indicated as ES (95%CI). The distribution of HLA-DRB1*0301 was inequality in the worldwide populations, European ancestry populations presented a higher allele frequency of HLA-DRB1*0301 than African and Latin American ancestry populations and Asians (0.114 Vs. 0.071, 0.069, 0.041). In intra-Asian, Taiwanese presented a higher allele frequency of HLA-DRB1*0301 than populations in USA, mainland China, and Korea(0.091 Vs.0.054, 0.047, 0.022). However, HLA-DRB1*0301 was a rare allele in Japanese.

**Figure 3:**
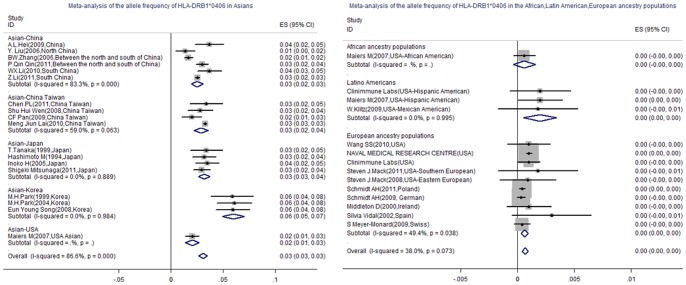
Forest plots summarizing the Allele frequency of HLA-DRB1*0406 in Asian and European ancestry populations from various regions. The allele frequency was indicated as ES (95%CI). The distribution of HLA-DRB1*0406 was inequality in the worldwide populations, it was common in Asians but rare in African, Latin American and European ancestry populations (0.031 Vs.0.001, 0.002, 0.001). In intra- Asian, Korean presented a higher allele frequency of HLA-DRB1*0406 than populations in Japan, China Taiwan, mainland China, and USA(0.060 Vs.0.032,0.029,0.025,0.021).

**Table 3:** The allele frequencies of HLA-DRB1*0301 and HLA-DRB1*0406 in populations from various ethnic regions.

Author	Ethnic origin	Geographic Region	Size	Allele	Frequency
A. L.Hei [25]	Asian	China(Beijing,Suzhou,Shenzhen)	718	0.0369	0.0369
Y. Liu [26]	Asian	North China(Beijing, Tianjin)	618	0.0348	0.0138
BW.Zhang [27]	Asian	^a.^North China(Henan province)	3874	0.0484	0.0169
P.Qin Qin [28]	Asian	^a.^Between the north and south	3238	0.0485	0.0298
WX.Li [29]	Asian	^a.^South China(Hubei province)	1013	0.0479	0.0370
Z.Li [30]	Asian	^a.^South China(Guangdong)	20621	0.0531	0.0254
Chen PL [31]	Asian	China Taiwan	504	0.0870	0.0340
ShuHui Wen [32]	Asian	^b.^China Taiwan	710	0.0880	0.0282
CF Pan [33]	Asian	China Taiwan	855	0.0740	0.0200
Meng Jiun	Asian	^b.^China Taiwan	46682	0.1103	0.0327
T.Tanaka [35]	Asian	^f.^Japan	525	0.0040	0.0340
Hashimoto	Asian	Japan	916	0.0020	0.0320
Inoko H [37]	Asian	Japan	1018	0.0010	0.0350
Shigeki	Asian	Japan	1616	0.0003	0.0297
M.H.Park [39]	Asian	Korea	510	0.0196	0.0588
M.H.Park [40]	Asian	Korea	800	0.0230	0.0610
Eun Young	Asian	Korea	800	0.0225	0.0594
Maiers M [42]	Asian	USA	1772	0.0537	0.0206
Maiers M [42]	African	USA	2411	0.0707	0.0006
Clinimmune	Hispanic American	^c.^USA	1058	0.0630	0.0020
Maiers M [42]	Hispanic American	USA	1999	0.0733	0.0020
W.Klitz [43]	Mexican American	USA	553	0.0678	0.0018
Wang SS [44]	European ancestry	USA	1070	0.1039	0.0010
NAVAL MEDICAL	European ancestry	^f.^USA	61655	0.1250	0.0010
Clinimmune	European ancestry	^c.^USA	3830	0.1210	0.0010
Steven	Southern European	USA	552	0.0987	0.0018
Steven	Eastern European	USA	558	0.1022	0.0009
Schmidt AH [47]	Eastern European	^d.^Poland	20653	0.1053	0.0004
Schmidt AH [48]	Western European	German	8862	0.1087	0.0003
Middleton D [49]	Western European	Ireland Northern	1000	0.1600	0.0010
Silvia Vidal [50]	Western European	^e.^Spain Barcelona	941	0.1200	0.0030
S Meyer	Western European	Swiss	3093	0.0972	0.0004

These data information were collected from the Allele Frequency Net Database [52] except reference 25,27–30,33–34,38–39,41,45,51. Allele Frequency: Total number of copies of the allele in the population sample (Alleles/2n) in decimal format. a: data from Chinese National Marrow Donor Program(CMDP), b: data from Tzu Chi Taiwan Marrow Donor Registry (TCTMDR), c: data from USA Colorado Univ. Cord Blood Bank, d: data from Poland DKMS, e: data from Umbilical Cord Blood Bank of Bacelona, f: data in Allele frequency net was calculated from Phenotype Frequencies assuming Hardy-Weinberg proportions.

### The Allele Frequencies of HLA-DRB1*0301 and HLA-DRB1*0406 in Populations from Various Ethnic Regions

To get the allele distribution of HLA-DRB1*0301 and HLA-DRB1*0406 in worldwide populations, we conducted a meta-analysis to summarize the allele frequencies of HLA-DRB1*0301 and HLA-DRB1*0406 in populations from various ethnic regions. A systemic literature review was made to identify 32 studies involving 195205 individuals met criteria for the meta analysis. (Table 3). The inclusion criteria was (i) randomised trial, no selection bias. (ii) subjects were all collected from healthy adult volunteers, and sample size ≥500. (iii) protocols for HLA-DRB1 genotyping: High-resolution genotyping analysis. (iv) confirm to Hardy-Weinberg equilibrium. (v) The allele frequencies were calculated by direct gene counting method. (vi) The allele frequencies of HLA-DRB1*0301 and HLA-DRB1*0406 were detected all together. The Excluding criteria was (i) the basic information (eg. race, region) of subjects was unclear. (ii) there was a limitation in subject collections (eg. gender, age, smoker) (iii) the calculation method for allele frequency was unspecified. Calculations were carried out using Stata-11.0 software (StataCorp, College Station, TX, USA). Using a meta-analysis, we found that (i) the distribution of HLA-DRB1*0301 and HLA-DRB1*0406 was inequality in populations from various ethnic regions: European ancestry populations presented a higher allele frequency of HLA-DRB1*0301 but a lower allele frequency of HLA-DRB1*0406 than Asians (0.114 Vs. 0.041, 0.001 Vs. 0.031, respectively). European ancestry populations presented a higher allele frequency of HLA-DRB1*0301 than African and Latin American ancestry populations (0.114 Vs. 0.071, 0.069), and HLA-DRB1*0406 was presented as a rare allele in these regions. (ii) In intra-Asians: Taiwanese presented a higher allele frequency of HLA-DRB1*0301 than populations from USA, mainland China, and Korea (0.091Vs.0.054, 0.047, 0.022), However, HLA-DRB1*0301 was presented as a rare allele in Japanese. Korean presented a higher allele frequency of HLA-DRB1*0406 than populations from Japan, China Taiwan, mainland China and USA (0.060 Vs.0.032, 0.029, 0.025, 0.021). (Figure 2&3).

**Table 4:** The tag SNPs of HLA-DRB1 alleles in four HapMap populations.

HLA-DRB1 alleles	CHB	JPT	CEU	YRI
**0301**	rs5000803	–	rs2040410	rs2040410
	rs3129299	–	rs2187688	rs9277489
	rs7769979	–	–	rs9275229
**0401**	–	rs1150758	–	rs3129763
**0403**	rs2075800	rs169494	rs7454108	–
	rs2395175	rs3763349	rs206765	–
	–	–	rs399604	–
**0406**	–	rs2395175	–	–
**0701**	rs3129859	rs6936863	rs7745002	–
**0802**	–	rs3129888	–	–
**0803**	rs3129859	rs2395148	–	–
**0901**	rs2395185	rs2187818	–	–
**1201**	–	–	–	rs4248166
**1202**	rs6916742	rs4418214	–	–
	rs646984	–	–	–
**1301**	rs4947342	–	rs2395173	rs3134942
**1302**	rs11758998	rs6936204	rs4434496	rs2157339
**1401**	–	–	–	rs1612904
**1406**	–	rs3129888	–	–
**1501**	rs7773756	–	–	–
**1502**	rs2858880	rs3135365	rs2858880	–

Tag SNPs were obtained from the references [75]–[80].“−”: the tag SNP has not been detected. rs9275229 has merged into rs2858324. CHB: Han Chinese in Beijing, JPT:Japanese in Tokyo,CEU: Utah Residents with Northern and Western European Ancestry, YRI:Yoruba in Ibadan, Nigeria.

## Discussion

In this study, we found that HLA-DRB1 alleles may be contributing to susceptibility to sporadic PD in the Chinese Han population in the Guangdong Province of PRC. The allele frequency of HLA-DRB1*03 was significantly higher in PD patients than in healthy controls (0.0811 vs. 0.0449, pc = 1.789E-3, statistic power >0.75), which was identical with the report in European ancestry populations [16]. Furthermore, we found that HLA-DRB1*0301, the most common subtype of HLA-DRB1*03, had a strong association with PD development.

HLA-DRB1*0301 has been reported contribute to the genetic susceptibility of some autoimmune diseases such as type 1 diabetes and multiple sclerosis [53]–[54]. In our study, the allele frequency of HLA-DRB1*0301 was significantly higher in PD patients than in healthy controls (0.0811 vs. 0.0449, pc = 4.572E-3, statistic power >0.75). Furthermore, the allele frequency was significantly higher in patients with an onset age ≤50 than in patients with an onset age >50(0.0678 vs. 0.0342, *pc = 0.012,* statistic Power >0.75), suggesting that people carrying the HLA-DRB1*0301 allele may have a tendency for an early onset of PD. Molecules encoded by polymorphic HLA alleles elicit different T-cell killing effects by presenting different peptide-binding preferences [55]. In pulmonary sarcoidosis, patients carrying the HLA-DRB1*0301 allele have shown more AV2S3 positive T-cells, but less regulatory T-cells than those carrying other HLA-DRB1 alleles [56]–[57]. Interestingly, several studies reported that regulatory T-cells could mediate neuroprotection through modulation of microglia oxidative stress and inflammation, while profound T-cell responses could exacerbate neuroinflammation and induce dopaminergic neurodegeneration [58]–[61]. Thus, we speculated that the HLA-DRB1*0301 allele may be related to younger patients through eliciting strong inflammatory responses.

The HLA-DRB1 *0406 allele, a subtype of HLA-DRB1*04, whether play a role to prevent the development of sporadic PD deserves debate. The HLA-DRB1*0406 allele is rare in European ancestry populations, but common in Asian ancestry populations [25]–[52]. Previous studies have shown that it is associated with susceptibility to insulin autoimmune syndrome, silicosis, prostate cancer, and pemphigus in Asian ancestry populations [62]–[65], which implied that HLA-DRB1*0406 is a risk allele for immune dysfunction diseases. In our study, the allele frequency of HLA-DRB1*0406 in PD patients was significantly lower than in healthy controls (0.0013 vs.0.0308, pc = 3.295E-5, statistic Power >0.75) in series 1, but the difference was not achieve statistical significance in series 2 (0.0078 vs. 0.0267, p = 0.097, pc = 1), this inconsistency may be due to a sampling bias. We noticed that the allele frequencies of HLA-DRB1*0403, another subtype of HLA-DRB1*04, was presented higher in PD patients than in healthy controls in series 1 although the difference was not significant after Bonferroni adjustment (0.0307 vs. 0.0159, p = 0.035, pc = 1). There was no report that HLA-DRB1*04 have two opposing effects in the disease. Therefore, whether HLA-DRB1*0406 plays a unique neuroprotective role in the PD development of Chinese Han population still needs to be investigated.

After summarizing the allele frequencies of HLA-DRB1*0301 and HLA-DRB1*0406 in populations from various ethnic regions, we confirmed that the distribution of the two HLA-DRB1 alleles was highly polymorphic with different ethnic and geographic, this phenomenon was more obviously in vast and complicated races regions, such as mainland China, China Taiwan, and America. We also found a higher frequency of HLA-DRB1*0301, but a lower frequency of HLA-DRB1*0406 in European ancestry populations than in Asian ancestry populations. This was consistent with the higher prevalence of PD in European ancestry populations than in Asian ancestry populations [66]–[69]. However, we could not analyze the correlation between the allele frequencies of these loci and prevalence of sporadic PD because the allele frequency data was incomplete and the prevalence data was collected using different methodologies in the worldwide population.

We noticed that the allele frequencies of HLA-DRB1*1202, DRB1*1312, and DRB1*1454 were also higher in PD patients than in healthy controls, However, the difference was not significant after Bonferroni adjustment, the statistic power in those alleles are at moderate level (0.5< statistic Power <0.75). Several studies reported these alleles unequivocally associated with immune-related diseases [70]–[74]. The possibility cannot be ruled out that these alleles may be either susceptibility genes or linkage disequilibrium with other susceptibility genes contributing to the etiology of PD. In addition, some rare alleles were detected because of random sampling, we lost most of the statistical power in these alleles due to their very small allele frequencies.

Recent studies have shown that some common HLA alleles can be marked with SNP-based tags [75]–[80]. We listed tag SNPs of the HLA-DRB1 alleles (Table 4), and compared it with the reported genetic susceptibility SNPs in the HLA-DR region to sporadic PD in European ancestry populations. We found that (i) these tag SNPs are all located at a considerable distance from the HLA-DRB1 allele. (ii) tag SNPs are likely to differ between populations, HLA-DRB1*0406 in JPT(Japanese in Tokyo) and HLA-DRB1*0403 in CHB(Han Chinese in Beijing) shared the same tag SNP:rs2395175, our study showed that HLA-DRB1*0403 was more common in PD patients than in controls, but the difference was no statistic significance, that partially explained why PD GWAS study in Japanese saw no evidence of HLA association even though the HLA-DRB1*0406 allele appears more frequent in Japan [20]. (iii) rs3129859 (tag HLA-DRB1*0701 in CHB), rs3129888(tag HLA-DRB1*0802,-DRB1*1406 in JPT), and rs3763313(tag HLA-DRB1*0803 in JPT) have been reported in association with the genetic susceptibility to PD in European ancestry populations [17]–[19], but there was no evidence showed that HLA-DRB1*0701, -DRB1*0802, -DRB1*0803,-DRB1*1406 were susceptibility loci to PD. The inconsistency may be arising from highly ethnic and region differences in HLA allele and SNP frequencies. In addition, reports on map of HLA alleles and its tag SNPs were few.

In conclusion, our study indicated that HLA-DRB1 alleles are associated with sporadic PD in a Chinese Han population, further research will be required to explore the role of HLA-DRB1 alleles in the pathogenesis of Parkinson’ disease.

## References

[1] Lang AE, Lozano AM (1998) Parkinson's disease. Second of two parts. N Engl J Med 339: 1130–1143. 9770561 10.1056/NEJM199810153391607

[2] Monahan AJ, Warren M, Carvey PM (2008) Neuroinflammation and peripheral immune infiltration in Parkinson's disease: an autoimmune hypothesis. Cell Transplant 17: 363–372. 18522239

[3] Ros-Bernal F, Hunot S, Herrero MT, Parnadeau S, Corvol JC, et al (2011) Microglial glucocorticoid receptors play a pivotal role in regulating dopaminergic neurodegeneration in parkinsonism. Proc Natl Acad Sci U S A 108: 6632–6637. 21467220 10.1073/pnas.1017820108 PMC3080980

[4] Yong J, Lacan G, Dang H, Hsieh T, Middleton B, et al (2011) BCG vaccine-induced neuroprotection in a mouse model of Parkinson's disease. PLoS One 6: e16610. 21304945 10.1371/journal.pone.0016610 PMC3031604

[5] Mcgeer PL, Itagaki S, Boyes BE, Mcgeer EG (1988) Reactive microglia are positive for HLA-DR in the substantia nigra of Parkinson's and Alzheimer's disease brains. Neurology 38: 1285–1291. 3399080 10.1212/wnl.38.8.1285

[6] Klein J, Sato A (2000) The HLA system. First of two parts. N Engl J Med 343: 702–709. 10974135 10.1056/NEJM200009073431006

[7] Handunnetthi L, Ramagopalan SV, Ebers GC, Knight JC (2010) Regulation of major histocompatibility complex class II gene expression, genetic variation and disease. Genes Immun 11: 99–112. 19890353 10.1038/gene.2009.83 PMC2987717

[8] Zota V, Nemirovsky A, Baron R, Fisher Y, Selkoe DJ, et al (2009) HLA-DR alleles in amyloid beta-peptide autoimmunity: a highly immunogenic role for the DRB1*1501 allele. J Immunol 183: 3522–3530. 19675171 10.4049/jimmunol.0900620

[9] Ma SL, Tang NL, Tam CW, Lui VW, Suen EW, et al (2008) Association between HLA-A alleles and Alzheimer's disease in a southern Chinese community. Dement Geriatr Cogn Disord 26: 391–397. 18936542 10.1159/000164275

[10] Guerini FR, Tinelli C, Calabrese E, Agliardi C, Zanzottera M, et al (2009) HLA-A*01 is associated with late onset of Alzheimer's disease in Italian patients. Int J Immunopathol Pharmacol 22: 991–999. 20074462 10.1177/039463200902200414

[11] Neill D, Curran MD, Middleton D, Mawhinney H, Edwardson JA, et al (1999) Risk for Alzheimer's disease in older late-onset cases is associated with HLA-DRB1*03. Neurosci Lett 275: 137–140. 10568518 10.1016/s0304-3940(99)00761-2

[12] Marttila RJ, Rinne UK, Tiilikainen A (1981) Histocompatibility types in Parkinson's disease. J Neurol Sci 51: 217–221. 6944440 10.1016/0022-510x(81)90100-3

[13] Leheny WA, Davidson DL, Devane P, House AO, Lenman JA (1983) HLA antigens in Parkinson's disease. Tissue Antigens 21: 260–261. 6602398 10.1111/j.1399-0039.1983.tb00167.x

[14] Reed E, Lewison A, Mayeaux R, Suciu-Foca N (1983) HLA antigens in Parkinson's disease. Tissue Antigens 21: 161–163. 6405503 10.1111/j.1399-0039.1983.tb00384.x

[15] Lampe JB, Gossrau G, Herting B, Kempe A, Sommer U, et al (2003) HLA typing and Parkinson's disease. Eur Neurol 50: 64–68. 12944708 10.1159/000072500

[16] Saiki M, Baker A, Williams-Gray CH, Foltynie T, Goodman RS, et al (2010) Association of the human leucocyte antigen region with susceptibility to Parkinson's disease. J Neurol Neurosurg Psychiatry 81: 890–891. 20462916 10.1136/jnnp.2008.162883

[17] Hamza TH, Zabetian CP, Tenesa A, Laederach A, Montimurro J, et al (2010) Common genetic variation in the HLA region is associated with late-onset sporadic Parkinson's disease. Nat Genet 42: 781–785. 20711177 10.1038/ng.642 PMC2930111

[18] Nalls MA, Plagnol V, Hernandez DG, Sharma M, Sheerin UM, et al (2011) Imputation of sequence variants for identification of genetic risks for Parkinson's disease: a meta-analysis of genome-wide association studies. Lancet 377: 641–649. 21292315 10.1016/S0140-6736(10)62345-8 PMC3696507

[19] Simón-Sánchez J, van Hilten JJ, van de Warrenburg B, Post B, Berendse HW, et al (2011) Genome-wide association study confirms extant PD risk loci among the Dutch. Eur J Hum Genet (19) 655–661. 10.1038/ejhg.2010.254 PMC3110043 21248740

[20] Satake W, Nakabayashi Y, Mizuta I, Hirota Y, Ito C, et al (2009) Genome-wide association study identifies common variants at four loci as genetic risk factors for Parkinson's disease. Nat Genet 41: 1303–1307. 19915576 10.1038/ng.485

[21] Marsh SG (2012) WHO Nomenclature Committee for Factors of the HLA System (2012) Nomenclature for factors of the HLA system, update December 2011. Tissue Antigens 79: 230–234. 22309261 10.1111/j.1399-0039.2012.01841.x

[22] Hughes AJ, Daniel SE, Kilford L, Lees AJ (1992) Accuracy of clinical diagnosis of idiopathic Parkinson's disease: a clinico-pathological study of 100 cases. J Neurol Neurosurg Psychiatry 55: 181–184. 1564476 10.1136/jnnp.55.3.181 PMC1014720

[23] Creary LE, Girdlestone J, Zamora J, Brown J, Navarrete CV (2009) Molecular typing of HLA genes using whole genome amplified DNA. Transfusion 49: 57–63. 18954395 10.1111/j.1537-2995.2008.01943.x

[24] Shi YY, He L (2005) SHEsis, a powerful software platform for analyses of linkage disequilibrium, haplotype construction, and genetic association at polymorphism loci. Cell Res 15: 97–98. 15740637 10.1038/sj.cr.7290272

[25] Hei AL, Li W, Deng ZH, He J, Jin WM, et al (2009) Analysis of high-resolution HLA-A, -B, -Cw, -DRB1, and -DQB1 alleles and haplotypes in 718 Chinese marrow donors based on donor–recipient confirmatory typings. Int J Immunogenet 36: 275–282. 19674163 10.1111/j.1744-313X.2009.00866.x

[26] Deng YJ, Yang G, Wu DY, Hu SN, Li SB, et al (2006) HLA-A, B, DRB1 gene polymorphism of Beijing population was studied by high-resolution polymerase chain reaction sequence-based typing. Zhonghua Yi Xue Yi Chuan Xue Za Zhi 23: 103–106. 16456803

[27] Zhang BW, Xing PQ, Guo RH, Bie LL, Zhao L, et al (2006) Study on the polymorphism of HLA genes in the stem cell donors of Henan province. Chinese journal of blood transfusion 19: 115–117.

[28] Qin Qin P, Su F, Xiao Yan W, Xing Z, Meng P, et al (2011) Distribution of human leucocyte antigen-A, -B and -DR alleles and haplotypes at high resolution in the population from Jiangsu province of China. Int J Immunogenet 38: 475–481. 21816002 10.1111/j.1744-313X.2011.01029.x

[29] Li WX, Shen G, Wu JM, Zhu YY, Liu GJ, et al (2010) The HLA-DRB1 gene polymorphism at high resolution of Han population from Hubei Branch of CMDP. Chinese journal of blood transfusion 23: 443–445.

[30] Huang CX, Cheng LH, Liu YZ, Li Z (2011) Study on the association between HLA-DRB1 allele polymorphism and the genetic susceptibility with Breast Cancer. International Journal of laboratory medicine 32: 728–733.

[31] Chen PL, Fann CS, Chu CC, Chang CC, Chang SW, et al (2011) Comprehensive genotyping in two homogeneous Graves' disease samples reveals major and novel HLA association alleles. PLoS One 6: e16635. 21307958 10.1371/journal.pone.0016635 PMC3030609

[32] Wen SH, Lai MJ, Yang KL (2008) Human leukocyte antigen-A, -B, and -DRB1 haplotypes of cord blood units in the Tzu Chi Taiwan Cord Blood Bank. Hum Immunol. 69: 430–436. 10.1016/j.humimm.2008.05.012 18582515

[33] Pan CF, Wu CJ, Chen HH, Dang CW, Chang FM, et al (2009) Molecular analysis of HLA-DRB1 allelic associations with systemic lupus erythematous and lupus nephritis in Taiwan. Lupus 18: 698–704. 19502265 10.1177/0961203308101955

[34] Lai MJ, Wen SH, Lin YH, Shyr MH, Lin PY, et al (2010) Distributions of human leukocyte antigen-A, -B, and -DRB1 alleles and haplotypes based on 46,915 Taiwanese donors. Hum Immunol 71: 777–782. 20493227 10.1016/j.humimm.2010.05.013

[35] Tanaka T, Ohmori M, Yasunaga S, Ohshima K, Kikuchi M, et al (1999) DNA typing of HLA class II genes (HLA-DR, -DQ and -DP) in Japanese patients with histiocytic necrotizing lymphadenitis (Kikuchi's disease). Tissue Antigens 54: 246–253. 10519361 10.1034/j.1399-0039.1999.540305.x

[36] Hashimoto M, Kinoshita T, Yamasaki M, Tanaka H, Imanishi T, et al (1994) Gene frequencies and haplotypic associations within the HLA region in 916 unrelated Japanese individuals. Tissue Antigens 44: 166–173. 7839349 10.1111/j.1399-0039.1994.tb02375.x

[37] Itoh Y, Mizuki N, Shimada T, Azuma F, Itakura M, et al (2005) High-throughput DNA typing of HLA-A, -B, -C, and -DRB1 loci by a PCR-SSOP-Luminex method in the Japanese population. Immunogenetics 57: 717–729. 16215732 10.1007/s00251-005-0048-3

[38] Mitsunaga S, Homma Y, Narita A, Kashiwase K, Okudaira Y, et al (2011) Particular human leukocyte antigen alleles are associated with biochemical traits in the Japanese population. Hum Immunol 72: 566–570. 21510991 10.1016/j.humimm.2011.03.011

[39] Park MH, Kim HS, Kang SJ (1999) HLA-A,-B,-DRB1 allele and haplotype frequencies in 510 Koreans. Tissue Antigens 53: 386–390. 10323346 10.1034/j.1399-0039.1999.530412.x

[40] Song EY, Park H, Roh EY, Park MH (2004) HLA-DRB1 and -DRB3 allele frequencies and haplotypic associations in Koreans. Hum Immunol 65: 270–276. 15041167 10.1016/j.humimm.2003.12.005

[41] Song EY, Park S, Lee DS, Cho HI, Park MH (2008) Association of human leukocyte antigen-DRB1 alleles with disease susceptibility and severity of aplastic anemia in Korean patients. Hum Immunol 69: 354–359. 18571007 10.1016/j.humimm.2008.04.009

[42] Maiers M, Gragert L, Klitz W (2007) High-resolution HLA alleles and haplotypes in the United States population. Hum Immunol 68: 779–788. 17869653 10.1016/j.humimm.2007.04.005

[43] Klitz W, Gragert L, Maiers M, Tu B, Lazaro A, et al (2009) Four-locus high-resolution HLA typing in a sample of Mexican Americans. Tissue Antigens 74: 508–513. 19845916 10.1111/j.1399-0039.2009.01374.x PMC3485641

[44] Wang SS, Abdou AM, Morton LM, Thomas R, Cerhan JR, et al (2010) Human leukocyte antigen class I and II alleles in non-Hodgkin lymphoma etiology. Blood 115: 4820–4823. 20385791 10.1182/blood-2010-01-266775 PMC2890176

[45] Mack SJ, Tu B, Yang R, Masaberg C, Ng J, et al (2011) Human leukocyte antigen-A, -B, -C, -DRB1 allele and haplotype frequencies in Americans originating from southern Europe: contrasting patterns of population differentiation between Italian and Spanish Americans. Hum Immunol 72: 144–149. 20974205 10.1016/j.humimm.2010.10.017 PMC3045865

[46] Mack SJ, Tu B, Lazaro A, Yang R, Lancaster AK, et al (2009) HLA-A, -B, -C, and -DRB1 allele and haplotype frequencies distinguish Eastern European Americans from the general European American population. Tissue Antigens 73: 17–32. 19000140 10.1111/j.1399-0039.2008.01151.x PMC3495166

[47] Schmidt AH, Solloch UV, Pingel J, Baier D, Böhme I, et al (2011) High-resolution human leukocyte antigen allele and haplotype frequencies of the Polish population based on 20,653 stem cell donors. Hum Immunol 72: 558–565. 21513754 10.1016/j.humimm.2011.03.010

[48] Schmidt AH, Baier D, Solloch UV, Stahr A, Cereb N, et al (2009) Estimation of high-resolution HLA-A, -B, -C, -DRB1 allele and haplotype frequencies based on 8862 German stem cell donors and implications for strategic donor registry planning. Hum Immunol 70: 895–902. 19683023 10.1016/j.humimm.2009.08.006

[49] Middleton D, Williams F, Hamill MA, Meenagh A (2000) Frequency of HLA-B alleles in a Caucasoid population determined by a two-stage PCR-SSOP typing strategy. Hum Immunol 61: 1285–1297. 11163085 10.1016/s0198-8859(00)00186-5

[50] Vidal S, Morante MP, Moga E, Mosquera AM, Querol S, et al (2002) Molecular analysis of HLA-DRB1 polymorphism in north-east Spain. Eur J Immunogenet 29: 75–77. 11841495 10.1046/j.0960-7420.2001.00293.x

[51] Meyer-Monard S, Passweg J, Troeger C, Eberhard HP, Roosnek E, et al (2009) Cord blood banks collect units with different HLA alleles and haplotypes to volunteer donor banks: a comparative report from Swiss Blood stem cells. Bone Marrow Transplant 43: 771–778. 19060930 10.1038/bmt.2008.391

[52] Gonzalez-Galarza FF, Christmas S, Middleton D, Jones AR (2011) Allele frequency net: a database and online repository for immune gene frequencies in worldwide populations. Nucleic Acids Res 39: D913–919. 21062830 10.1093/nar/gkq1128 PMC3013710

[53] Manan H, Angham AM, Sitelbanat A (2010) Genetic and diabetic auto-antibody markers in Saudi children with type 1 diabetes. Hum Immunol 71: 1238–1242. 20858521 10.1016/j.humimm.2010.09.008

[54] de la Concha EG, Cavanillas ML, Cénit MC, Urcelay E, Arroyo R, et al (2012) DRB1*03: 01 haplotypes: differential contribution to multiple sclerosis risk and specific association with the presence of intrathecal IgM bands. PLoS One 7: e31018. 22363536 10.1371/journal.pone.0031018 PMC3281895

[55] Kaufman JF, Auffray C, Korman AJ, Shackelford DA, Strominger J (1984) The class II molecules of the human and murine major histocompatibility complex. Cell 36: 1–13. 6198089 10.1016/0092-8674(84)90068-0

[56] Idali F, Wikén M, Wahlström J, Mellstedt H, Eklund A, et al (2006) Reduced Th1 response in the lungs of HLA-DRB1*0301 patients with pulmonary sarcoidosis. Eur Respir J 27: 451–459. 16507843 10.1183/09031936.06.00067105

[57] Wikén M, Grunewald J, Eklund A, Wahlström J (2012) Multiparameter phenotyping of T-cell subsets in distinct subgroups of patients with pulmonary sarcoidosis. J Intern Med 271: 90–103. 21682779 10.1111/j.1365-2796.2011.02414.x

[58] Reynolds AD, Banerjee R, Liu J, Gendelman HE, Mosley RL (2007) Neuroprotective activities of CD4+CD25+ regulatory T cells in an animal model of Parkinson's disease. J Leukoc Biol 82: 1083–1094. 17675560 10.1189/jlb.0507296

[59] Benner EJ, Banerjee R, Reynolds AD, Sherman S, Pisarev VM, et al (2008) Nitrated alpha-synuclein immunity accelerates degeneration of nigral dopaminergic neurons. PLoS One 3: e1376. 18167537 10.1371/journal.pone.0001376 PMC2147051

[60] Brochard V, Combadière B, Prigent A, Laouar Y, Perrin A, et al (2009) Infiltration of CD4+ lymphocytes into the brain contributes to neurodegeneration in a mouse model of Parkinson disease. J Clin Invest 119: 182–192. 19104149 10.1172/JCI36470 PMC2613467

[61] Kebir H, Kreymborg K, Ifergan I, Dodelet-Devillers A, Cayrol R, et al (2007) Human TH17 lymphocytes promote blood-brain barrier disruption and central nervous system inflammation. Nat Med 13: 1173–1175. 17828272 10.1038/nm1651 PMC5114125

[62] Murakami M, Mizuide M, Kashima K, Kojima A, Tomioka SI, et al (2000) Identification of monoclonal insulin autoantibodies in insulin autoimmune syndrome associated with HLA-DRB1*0401. Horm Res 54: 49–52. 11182636 10.1159/000063437

[63] Ueki A, Isozaki Y, Kusaka M (2005) Anti-caspase-8 autoantibody response in silicosis patients is associated with HLA-DRB1, DQB1 and DPB1 alleles. J Occup Health 47: 61–67. 15703453 10.1539/joh.47.61

[64] Azuma H, Sada M, Tsuji T, Ueda H, Katsuoka Y (1999) Relationship between HLA-DR antigen and HLA-DRB1 alleles and prostate cancer in Japanese men. Int Urol Nephrol 31: 343–349. 10672954 10.1023/a:1007126219791

[65] Miyagawa S, Amagai M, Niizeki H, Yamashina Y, Kaneshige T, et al (1999) HLA-DRB1 polymorphisms and autoimmune responses to desmogleins in Japanese patients with pemphigus. Tissue Antigens 54: 333–340. 10551416 10.1034/j.1399-0039.1999.540402.x

[66] Zhang ZX, Roman GC, Hong Z, Wu CB, Qu QM, et al (2005) Parkinson's disease in China: prevalence in Beijing, Xian, and Shanghai. Lancet 365: 595–597. 15708103 10.1016/S0140-6736(05)17909-4

[67] von Campenhausen S, Bornschein B, Wick R, Bötzel K, Sampaio C, et al (2005) Prevalence and incidence of Parkinson's disease in Europe. Eur Neuropsychopharmacol 15: 473–490. 15963700 10.1016/j.euroneuro.2005.04.007

[68] Driver JA, Logroscino G, Gaziano JM, Kurth T (2009) Incidence and remaining lifetime risk of Parkinson disease in advanced age. Neurology 72: 432–438. 19188574 10.1212/01.wnl.0000341769.50075.bb PMC2676726

[69] Wirdefeldt K, Adami HO, Cole P, Trichopoulos D, Mandel J (2011) Epidemiology and etiology of Parkinson's disease: a review of the evidence. Eur J Epidemiol 26 Suppl 1 S1–58. 21626386 10.1007/s10654-011-9581-6

[70] Chun-Lai T, Padyukov L, Dhaliwal JS, Lundström E, Yahya A, et al (2011) Shared epitope alleles remain a risk factor for anti-citrullinated proteins antibody (ACPA)–positive rheumatoid arthritis in three Asian ethnic groups. PLoS One 6: e21069. 21698259 10.1371/journal.pone.0021069 PMC3115981

[71] Kojima Y, Takahara S, Nonomura N, Sada M, Tsuji T, et al (2000) HLA-DRB genotypes in Japanese patients with renal cell carcinoma. Oncology 59: 57–62. 10895068 10.1159/000012138

[72] Monos DS, Papaioakim M, Ho TW, Li CY, Mckhann GM (1997) Differential distribution of HLA alleles in two forms of Guillain- Barré syndrome. J Infect Dis 176 Suppl 2 S180–182. 9396707 10.1086/513786

[73] Luo H, Chen M, Yang R, Xu PC, Zhao MH (2011) The association of HLA-DRB1 alleles with antineutrophil cytoplasmic antibody-associated systemic vasculitis in Chinese patients. Hum Immunol 72: 422–425. 21354458 10.1016/j.humimm.2011.02.017

[74] Saha M, Harman K, Mortimer NJ, Binda V, Black MM, et al (2010) Pemphigus vulgaris in White Europeans is linked with HLA Class II allele HLA DRB1*1454 but not DRB1*1401. J Invest Dermatol 130: 311–314. 19847191 10.1038/jid.2009.241

[75] Miretti MM, Walsh EC, Ke X, Delgado M, Griffiths M, et al (2005) A high-resolution linkage-disequilibrium map of the human major histocompatibility complex and first generation of tag single-nucleotide polymorphisms. Am J Hum Genet 76: 634–646. 15747258 10.1086/429393 PMC1199300

[76] de Bakker PI, McVean G, Sabeti PC, Miretti MM, Green T, et al (2006) A high-resolution HLA and SNP haplotype map for disease association studies in the extended human MHC. Nat Genet 38: 1166–1172. 16998491 10.1038/ng1885 PMC2670196

[77] Leslie S, Donnelly P, McVean G (2008) A statistical method for predicting classical HLA alleles from SNP data. Am J Hum Genet 82: 48–56. 18179884 10.1016/j.ajhg.2007.09.001 PMC2253983

[78] Tekola Ayele F, Hailu E, Finan C, Aseffa A, Davey G, et al (2012) Prediction of HLA class II alleles using SNPs in an African population. PLoS One 7: e40206. 22761960 10.1371/journal.pone.0040206 PMC3386230

[79] McElroy JP, Isobe N, Gourraud PA, Caillier SJ, Matsushita T, et al (2011) SNP-based analysis of the HLA locus in Japanese multiple sclerosis patients. Genes Immun 12: 523–530. 21654846 10.1038/gene.2011.25 PMC3361962

[80] Kitajima H, Sonoda M, Yamamoto K (2012) HLA and SNP haplotype mapping in the Japanese population. Genes Immun 35. In press. 10.1038/gene.2012.35 22914434

